# Occupational Animal Exposure Among Persons with Campylobacteriosis and Cryptosporidiosis — Nebraska, 2005–2015

**DOI:** 10.15585/mmwr.mm6636a4

**Published:** 2017-09-15

**Authors:** Chia-ping Su, Derry T. Stover, Bryan F. Buss, Anna V. Carlson, Sara E. Luckhaupt

**Affiliations:** ^1^Epidemic Intelligence Service, CDC; ^2^Division of Surveillance, Hazard Evaluations, and Field Studies, National Institute for Occupational Safety and Health, CDC; ^3^Nebraska Department of Health and Human Services; ^4^Career Epidemiology Field Officer Program, CDC.

*Campylobacter* and *Cryptosporidium* are two common causes of gastroenteritis in the United States. National incidence rates measured for these pathogens in 2015 were 17.7 and 3.0 per 100,000 population, respectively; Nebraska was among the states with the highest incidence for both campylobacteriosis (26.6) and cryptosporidiosis (≥6.01) (*1*). Although campylobacteriosis and cryptosporidiosis are primarily transmitted via consumption of contaminated food or water, they can also be acquired through contact with live animals or animal products, including through occupational exposure (*2*). This exposure route is of particular interest in Nebraska, where animal agriculture and associated industries are an important part of the state’s economy. To estimate the percentage of disease that might be related to occupational animal exposure in Nebraska, the Nebraska Department of Health and Human Services (NDHHS) and CDC reviewed deidentified investigation reports from 2005 to 2015 of cases of campylobacteriosis and cryptosporidiosis among Nebraska residents aged ≥14 years. Case investigation notes were searched for evidence of occupational animal exposures, which were classified into discrete categories based on industry, animal/meat, and specific work activity/exposure. Occupational animal exposure was identified in 16.6% of 3,352 campylobacteriosis and 8.7% of 1,070 cryptosporidiosis cases, among which animal production (e.g., farming or ranching) was the most commonly mentioned industry type (68.2% and 78.5%, respectively), followed by employment in animal slaughter and processing facilities (16.3% and 5.4%, respectively). Among animal/meat occupational exposures, cattle/beef was most commonly mentioned, with exposure to feedlots (concentrated animal feeding operations in which animals are fed on stored feeds) reported in 29.9% of campylobacteriosis and 7.9% of cryptosporidiosis cases. Close contact with animals and manure in feedlots and other farm settings might place workers in these areas at increased risk for infection. It is important to educate workers with occupational animal exposure about the symptoms of enteric diseases and prevention measures. Targeting prevention strategies to high-risk workplaces and activities could help reduce disease.

After cases of campylobacteriosis or cryptosporidiosis are reported to the state, investigations are completed by surveillance staff members of local health departments, who contact patients and health care providers or use Electronic Medical Records to collect epidemiologic information, including the patient’s occupation. NDHHS and CDC analyzed deidentified reports for all confirmed and probable campylobacteriosis and cryptosporidiosis cases among Nebraska residents aged ≥14 years during 2005–2015 from the Nebraska Electronic Disease Surveillance System. Occupational animal exposure information was abstracted from free text investigation notes by searching all records for relevant keywords. For patients with occupational animal exposure, records were reviewed for type of work and then classified into four industry categories: 1) animal production, 2) animal slaughtering and processing, 3) veterinary services, and 4) other. The animal/meat types mentioned in the free text comments also were classified into four discrete categories: 1) cattle and other bovines, 2) chicken and other poultry, 3) swine, and 4) other or multiple farm animals. Several specific work activities and exposures among cattle production workers, including feedlot exposure, fecal exposure, hauling, and branding cattle were identified.

During 2005–2015, occupational animal exposure was identified among 557 (16.6%) of 3,352 residents of Nebraska aged ≥14 years with campylobacteriosis and 93 (8.7%) of 1,070 with cryptosporidiosis (Table 1). Among both campylobacteriosis and cryptosporidiosis cases, male and younger patients were more likely to have occupational animal exposure than female and older patients. Among campylobacteriosis and cryptosporidiosis cases with occupational animal exposure, 380 (68.2%) and 73 (78.5%) patients, respectively, reported animal production, and 91 (16.3%) and five (5.4%) patients, respectively, reported animal slaughtering and processing (Table 2). Cattle were the most common animal types mentioned among workers in both industries for both diseases. Among workers with campylobacteriosis, poultry and swine were the second and third most commonly reported animal types in both industries. Among cattle production workers, feedlot exposure, fecal exposure, hauling cattle, and branding cattle were reported by 29.9%, 8.9%, 6.6%, and 3.0% of campylobacteriosis patients, respectively, and by 7.9%, 11.1%, 6.3%, and 6.3% of cryptosporidiosis patients, respectively (Figure).

**TABLE 1:** Number and percentage of campylobacteriosis and cryptosporidiosis cases, by occupational animal exposure status and selected characteristics — Nebraska, 2005–2015

Characteristic	Campylobacteriosis (N = 3,352)			Cryptosporidiosis (N = 1,070)		
	Occupational animal exposure No. (%)	No occupational animal exposure No. (%)	p-value	Occupational animal exposure No. (%)	No occupational animal exposure No. (%)	p-value
**Total**	557 (16.6)	2,795 (83.4)	**—**	93 (8.7)	977 (91.3)	**—**
**Sex**						
Male	433 (22.0)	1,539 (78.0)	<0.01	57 (12.5)	401 87.6)	<0.01
Female	122 (8.9)	1,243 (91.1)		34 (5.6)	573 (94.4)	
Unknown	2 (13.3)	13 (86.7)		2 (40.0)	3 (60.0)	
**Age group (yrs)**						
14–24	143 (23.4)	468 (76.6)	<0.01	39 (15.8)	208 (84.2)	<0.01
25–34	128 (20.9)	484 (79.1)		22 (9.2)	218 (90.8)	
35–44	91 (18.5)	400 (81.5)		11 (6.3)	164 (93.7)	
45–54	89 (16.0)	468 (84.0)		12 (9.8)	111 (90.2)	
55–64	53 (10.8)	437 (89.2)		7 (6.3)	105 (93.8)	
≥65	53 (9.0)	538 (91.0)		2 (1.2)	171 (98.8)	
**Race/Ethnicity**						
White	236 (16.9)	1,163 (83.1)	0.01	37 (8.4)	405 (91.6)	0.21
Black	4 (17.4)	19 (82.6)		0 (—)	20 (100.0)	
Hispanic	20 (32.8)	41 (67.2)		0 (—)	20 (100.0)	
Other	10 (19.6)	41 (80.4)		0 (—)	13 (100.0)	
Unknown	287 (15.8)	1,531 (84.2)		56 (9.7)	519 (90.3)	
**Hospitalized**						
Yes	102 (15.2)	568 (84.8)	<0.01	22 (10.7)	184 (89.3)	0.19
No	431 (21.1)	1,610 (78.9)		63 (8.9)	649 (91.2)	
Unknown	24 (3.7)	617 (96.3)		8 (5.3)	144 (94.7)	
**Outcome**						
Died	0 (—)	9 (100.0)	<0.01	0 (—)	3 (100.0)	0.11
Survived	513 (20.2)	2,031 (79.8)		83 (9.6)	785 (90.4)	
Unknown	44 (5.5)	755 (94.5)		10 (5.0)	189 (95.0)	

**TABLE 2:** Number and percentage of campylobacteriosis and cryptosporidiosis patients who had occupational animal exposure, by industry and type of animal — Nebraska, 2005–2015

Industry (type of animal)	No. (%)	
	Campylobacteriosis	Cryptosporidiosis
**Total**	**557 (100.0)**	**93 (100.0)**
**Animal production**	**380 (68.2)**	**73 (78.5)**
(Cattle and other bovines)	271 (71.3)	63 (86.3)
(Chicken and other poultry)	35 (9.2)	0 (—)
(Swine)	21 (5.5)	0 (—)
(Other/Multiple farm animals)	53 (14.0)	10 (13.7)
**Animal slaughtering and processing**	**91 (16.3)**	**5 (5.4)**
(Beef cattle processing)	52 (57.1)	2 (40.0)
(Poultry processing)	13 (14.3)	0 (—)
(Swine processing)	10 (11.0)	2 (40.0)
(Multiple animals/Unspecified)	16 (17.6)	1 (20.0)
**Veterinary services**	**24 (4.3)**	**7 (7.5)**
**Other (shelter, rescue, pet store)**	**62 (11.1)**	**8 (8.6)**

**FIGURE:**
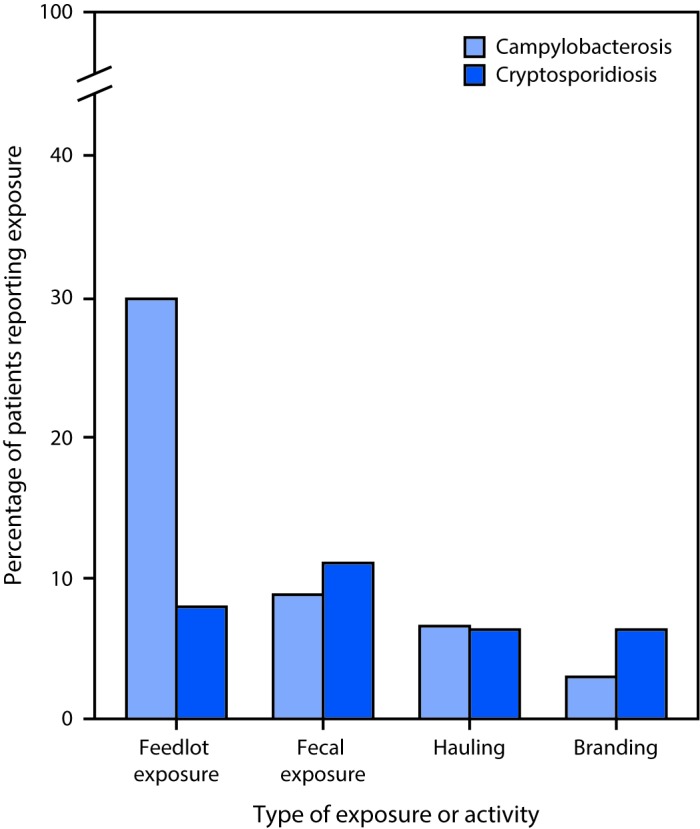
Percentage of campylobacteriosis (N = 557) and cryptosporidiosis (N = 93) patients with occupational cattle exposure in the animal production industry, by type of exposure or activity* — Nebraska, 2005–2015 * Patients might have more than one exposure or activity.

## Discussion

Although consumption of contaminated poultry and poultry products is known to be a common source of exposure to *Campylobacter* species (*3*), many other animals also can be infected, including cattle, and infection can be acquired through contact with live animals or contaminated meat. Whereas cryptosporidiosis outbreaks often are associated with contaminated recreational water (*4*), *Cryptosporidium* infections in calves occur commonly, and outbreaks resulting from animal-to-person transmission have been reported (*5*). This report describes occupational animal exposure, including the type of animal, workplace, and activity, among campylobacteriosis and cryptosporidiosis patients in an agricultural state during 2005–2015. One possible explanation for the high incidence rates of these infections in Nebraska is a high rate of exposure to livestock. There were an estimated 6.5 million head of cattle and calves in Nebraska in 2017,* which is 3.4 times more than the state’s population of 1.9 million persons.^†^ The overall rate for the United States is 0.3 head of cattle and calves per person.^§^

Workers in all animal-related industries, including animal production, animal slaughtering and processing, and other supportive services are at risk for zoonotic enteric diseases because of their daily and long-term exposure to live animals or animal products. Contact with farm animals and animal feces have been identified through a case-control study as risk factors for sporadic *Campylobacter* infection in the United States (*3*). Research has suggested that zoonotic transmission might be frequently associated with sporadic cryptosporidiosis cases (*6*) and that agricultural workers have increased potential for contracting various bovine zoonotic infections (*7*); in 2011, a cluster of *Campylobacter* infections was reported among persons working at a sheep ranch (*8*). Clusters of occupationally acquired cryptosporidiosis also have been reported among veterinary students, firefighters who responded to a fire on a cattle farm, and emergency responders attending to the rollover of a truck carrying calves (*5*). However, the proportion of cases of specific enteric diseases with occupational animal exposure has not been well characterized because occupational information is not universally collected in current infectious disease surveillance systems. In addition, when occupational information is collected, it is usually not recorded in standardized or discrete fields, often precluding data abstraction and analysis.

In this analysis, Nebraska feedlots, farms, and ranches were the most common workplace exposure settings for campylobacteriosis and cryptosporidiosis. Cattle were the animal type most commonly mentioned by patients with both conditions who had occupational animal exposure. Several specific activities and exposures in these workplace settings were mentioned in the investigation reports, including fecal exposure, hauling, and branding cattle. Close contact with animals and manure in feedlots and other farm settings where cattle are more concentrated might place workers in these areas at increased risk for infection. Studies have shown that prevalence of *Campylobacter* in feedlot cattle increases throughout the feeding period (*9*). In addition to having direct exposure, exposed workers might also carry pathogens beyond the workplace, placing family members or other close contacts at risk for exposure and illness.

Beyond on-farm exposures, cases of both campylobacteriosis and cryptosporidiosis were also reported among workers in animal slaughtering and processing facilities in Nebraska. Campylobacteriosis has been previously reported among workers at poultry processing plants, which are known to have a high potential for contamination with *Campylobacter* (*10*). However, most cases reported in Nebraska had occupational animal exposure through cattle slaughtering and processing, which is more prevalent in the state than poultry processing.

The findings in this report are subject to at least three limitations. First, it is not possible to infer causation from reported occupational animal exposure. Other possible exposure sources were not evaluated in this analysis. Second, because occupational animal exposure information was collected only if a patient volunteered such information or if an investigator asked for it informally, these estimates likely are conservative, and the actual proportion of ill persons having occupational animal exposures remains unknown. Finally, standardization of data collection was not emphasized among staff members who completed the interviews and investigations in multiple local health departments. As a result, misclassification and underestimation might have occurred despite use of a consistent process to manually review and classify cases.

This report describes types and percentages of occupational animal exposures among campylobacteriosis and cryptosporidiosis patients in Nebraska, which could represent important disease transmission routes in an agricultural state and have not been reported previously. Studies specifically focusing on pathogen transmission between animals and workers are needed to clarify the role of occupational animal contact in such diseases and identify effective strategies to minimize occupational risk. It is important that workers with occupational animal exposure be educated about symptoms of diseases and preventive measures, which include using dedicated clothing at work and proper handwashing after touching animals.^¶^ Routine collection of information on occupation via infectious disease surveillance systems could improve capture of data to ascertain the extent of occupationally acquired disease and establish causation. Regular review by employers and public health professionals of all cases of illness among animal industry workers in order to detect the potential for workplace acquisition could help in planning interventions to promote workers’ health.

**Summary**

**What is already known about this topic?**

Campylobacteriosis and cryptosporidiosis are two common causes of gastroenteritis, with incidence rates of 26.6 and ≥6.01 per 100,000 population in Nebraska, respectively. Although campylobacteriosis and cryptosporidiosis are primarily transmitted via consumption of contaminated food or water, they can also be acquired through contact with live animals or animal products, exposures which can be occupational.

**What is added by this report?**

During 2005–2015, occupational animal exposure was identified in 557 of 3,352 (16.6%) campylobacteriosis and 93 of 1,070 (8.7%) cryptosporidiosis cases in Nebraska in persons aged ≥14 years. Animal production (e.g., farming or ranching) was the most common type of industry among patients with occupational animal exposure, and cattle were the most commonly mentioned animal.

**What are the implications for public health practice?**

It is important that workers with occupational animal exposure be educated about symptoms of enteric diseases and prevention measures, which include using dedicated clothing at work and proper handwashing after touching animals. Routine collection of information on occupation in dedicated fields in infectious disease surveillance systems could improve the use of data to ascertain the extent of occupationally acquired disease and protect workers’ health.

## Footnotes

*
http://www.nda.nebraska.gov/facts.pdf .

† https://factfinder.census.gov/faces/tableservices/jsf/pages/productview.xhtml?pid=PEP_2016_PEPANNRES&src=pt.

§ According to the National Agricultural Statistics Service (https://www.nass.usda.gov/Charts_and_Maps/Cattle/), there were 93,584,600 head of cattle and calves in the United States. The U.S. population estimate was 323,127,513 in 2016.

¶ http://umash.umn.edu/wp-content/uploads/2015/11/Cryptosporidium-MDH.pdf and http://umash.umn.edu/wp-content/uploads/2015/11/Campylobacter-MDH.pdf.
